# PDA-associated infective endocarditis with pulmonary artery perforation

**DOI:** 10.12669/pjms.41.1.10200

**Published:** 2025-01

**Authors:** Fatina Munawar, Ikram Ahmed Rana, Muhammad Ali Mumtaz

**Affiliations:** 1 Fatina Munawar, MBBS. Tahir Heart Institute, Fazl-e-Omar Hospital, Chenab Nagar, District Chiniot, Pakistan; 2 Ikram Ahmed Rana, MBBS, FCPS. Tahir Heart Institute, Fazl-e-Omar Hospital, Chenab Nagar, District Chiniot, Pakistan; 3 Muhammad Ali Mumtaz, MD FACS. Tahir Heart Institute, Fazl-e-Omar Hospital, Chenab Nagar, District Chiniot, Pakistan

**Keywords:** Patent ductus arteriosus, Infective endocarditis, Pulmonary artery, Circulatory arrest

## Abstract

Infective endocarditis used to frequently cause mortality in subjects having PDA before the advent of antibiotics and surgical ligation. It has been documented that clinically silent PDAs may cause infective complications of heart valves. We present case of an 18-years-old male who presented with palpitations and fever to our emergency department. The fever was sudden in onset, associated with rigors, high-grade and with a continuous pattern for 12 days. The patient was previously managed as a case of dengue fever based on serology. Eight days after the onset of fever, the patient developed left-sided chest pain. Past medical record showed documentation of a patent ductus arteriosus.

A two-dimensional echocardiography showed circumferential pericardial effusion and a small-sized PDA; the left ventricular function was normal. Nevertheless, the definitive cause of the pericardial effusion was not known. The patient became haemodynamically unstable during the hospital stay and it was planned to ligate the PDA with cardiac surgical consultation. A left thoracotomy approach was chosen for the PDA ligation that unveiled rupture of the main pulmonary artery, so the pulmonary artery repair alongside the PDA ligation was planned with median sternotomy approach and cardiopulmonary bypass immediately. The patient had a 14-days course of antibiotics during the ICU stay. In conclusion, infective endocarditis remains a rare yet life-threatening complication of PDA irrespective of the size; a timely PDA-ligation could prevent the life-threatening sequels.

## INTRODUCTION

It has been documented that one in two thousand neonates has patent ductus arteriosus; moreover, in premature neonates there is an increased incidence of PDA totaling up to 20% - 60%.^1^ Infective endarteritis has been a frequent cause of mortality in subjects having PDA with a 0-45% yearly incidence before the advent of antibiotics and surgical ligation; additionally, a PDA may lead to left or right heart failure.^2^ A review of related research also shows evidence of infective endocarditis in association with clinically silent PDA.^3^-^5^ We present case of an 18-years-old male with a past medical history of small PDA, who presented to the emergency department with palpitations and fever.

## CASE REPORT

An 18-years-old male presented with fever for 12 days: the fever was sudden in onset, high-grade, of continuous pattern and associated with rigors. The patient was initially managed as a presumed case of dengue fever based on serology: positive Ig M antibody and negative Ig G antibody. Eight days later, the patient developed left-sided chest pain that used to get aggravated on lying flat and somewhat relieved on leaning forward. There was no history of burning micturition, diarrhoea, productive cough or headache. Past medical record mentioned PDA. There was also history of contact with an active tuberculosis patient, his grandfather, in the past. The patient had a blood pressure of 140/80 mm Hg at presentation, regular pulse at 140 bpm, temperature of 101 degree Fahrenheit, respiratory rate of 20/minute and an oxygen saturation of 90% at room air.

General physical examination showed notable pallor. There were no puncture marks suggestive of possible intravenous drug abuse. There was no jugular venous distension or engorged neck veins. On examination of the precordium, apex beat was not shifted. There was a machinery murmur, at left 2^nd^ intercostal space; however, there was no pericardial rub. On auscultation of the back of chest, there was normal vesicular breathing with decreased air entry at left basal area. The systemic examination was unremarkable other than that.

**Fig.1:**
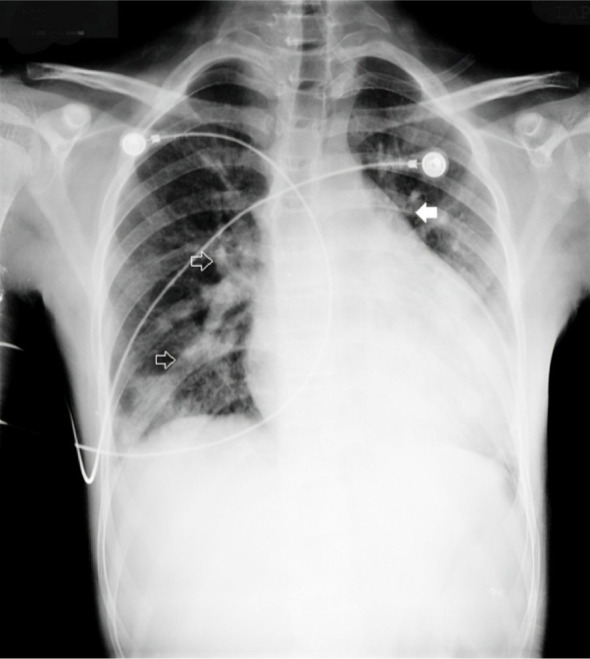
Chest X ray posterior-anterior view: a) increased cardiothoracic ratio, with somewhat prominent b) pulmonary artery marking on the left side (solid arrow); c) irregular-shaped opacities in the lung fields bilaterally (arrow outlines).

The initial ECG showed sinus tachycardia with concave ST-elevation and PR depression in leads I, II, aVL, V2. There was reciprocal ST depression and PR elevation in lead aVR. Laboratory investigations on day one showed a haemoglobin of 12.1 g/dl with a raised white cell count of 17 X 10^9/L. The ESR was also raised at 60 mm/hour. The blood cultures showed growth of methicillin-resistant staphylococcus aureus. The serological tests showed dengue Ig M positivity; however, the tuberculosis ICT was negative. Chest X ray posterior-anterior view (Fig.1) showed increased cardiothoracic ratio, with somewhat prominent pulmonary artery marking on the left side. There were irregular-shaped opacities in the lung fields bilaterally. The two-dimensional echocardiography showed circumferential pericardial effusion and a small-sized PDA. The left ventricular function was normal. Nevertheless, the definitive cause of the pericardial effusion was not known.

**Fig.2:**
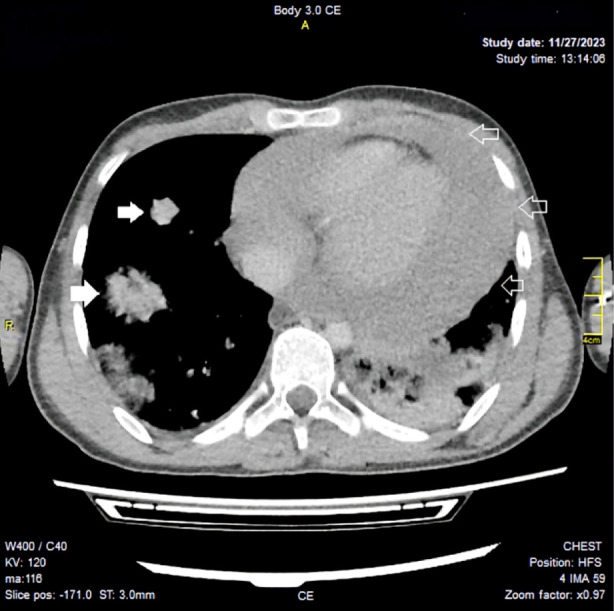
The CT chest: a) infiltrates in the lung fields (solid arrows); b) pericardial effusion (arrow outlines).

Approximately 12 hours after the admission, the patient developed acute shortness of breath associated with chest pain and apprehension on the night of the first day of admission. Blood pressure was 90/60 with heart rate of 150/minute. We suspected impending cardiac tamponade and cardiac surgeon was consulted. Pericardial window was planned together with PDA ligation. It was speculated to be infective endocarditis in association with patent ductus arteriosus. The CT chest (Fig.2) showed presence of infiltrates in the lung fields. Infective endocarditis in association with PDA was considered a possibility due to the presence of one major criterion and three minor criteria: the Duke’s criteria.

The blood cultures showed growth (major criterion) and there was fever >38 degree C, a predisposing cardiac lesion and vascular phenomena (septic emboli in lung fields). After consultation with cardiac surgery the initial plan was to do a left thoracotomy for PDA ligation and pericardial window to drain what was presumed to be purulent pericarditis. Upon drainage of the clotted blood in the pericardium, a perforation was discovered in the main pulmonary artery. The bleeding was controlled with finger pressure.

This resulted in a difficult situation where CPB was required during a left thoracotomy. Initially, the left femoral vessels were exposed, however the femoral artery was found to be too small to accept an adequate sized femoral cannula. Hence, the perforation was tightly packed with gauze and the pericardium oversewn to re-create partial tamponade of the perforation. The patient was flipped to supine position from the right decubitus position. CPB was initiated via median sternotomy with aorto-atrial cannulation. At 26 degree C, and under brief intermittent periods of circulatory arrest lasting less than three minutes the PDA was overseen from inside the pulmonary artery. Perforation was debrided and repaired with fresh autologous pericardium. It was inferred that infective endocarditis had led to perforation of the main pulmonary artery, resulting in haemopericardium. Intravenous antibiotics were continued for 14 days in the cardiothoracic surgical intensive care unit and the patient was discharged afterwards on oral linezolid for four additional weeks. At follow-up the patient is doing well off antibiotics with normal cardiac function.

## DISCUSSION

Callegari A, et al. concluded in a review article that PDA-IE has become an obsolete condition; however, with regards to the fatal complications of the condition, neonates having sepsis should be screened by means of a transthoracic echocardiography in case of pyrexia of unknown aetiology and signs such as a new heart murmur on auscultation of precordium.^3^ The most frequent manifestations of PDA-associated complications in adults are left heart failure and pulmonary hypertension; infective endocarditis has become an infrequent cause of morbidity in patients having PDA.^4^ Ozkokeli M, et al. presented a case of a 27-years-old woman having pulmonary and aortic valve endocarditis in association with a patent ductus arteriosus; it was a clinically silent PDA that was discovered on intervention after the median sternotomy was performed. This is similar to the case presented here, as the severity of disease and the aetiology of symptoms, i.e rupture of pulmonary artery was found out during surgical intervention; in our case, during left thoracotomy for the plan of PDA ligation.^5^

Typically, an uncomplicated PDA is ligated via a left thoracotomy or can be closed with a device using a percutaneous approach. Complicated PDA with endocarditis, calcification or aneurysm formation may require median sternotomy with cardiopulmonary bypass with or without circulatory arrest.^6^ In the case a thoracotomy approach is taken and CPB need is realized, this can be instituted via a femoral cut-down approach or a left atrial to subclavian artery approach. Due to a need to hold finger pressure, it would have been technically difficult to cannulate via a left thoracotomy. Furthermore, with the left atrial to subclavian artery approach, pulmonary arterial repair would have not been possible. Had the femoral vessels been adequate, a trans-femoral access for CPB would have been possible.

Fortunately, we were able to re-create tamponade on the main pulmonary artery which allowed us to do a median sternotomy and provide adequate CPB support for the required repair. Interestingly, no vegetations were noted on the pulmonary valve. The only infected area was that of the perforation. This was easily debrided and repaired. It has been shown that two-dimensional echocardiography has been a good diagnostic tool for infective endocarditis; nevertheless, the finding of pulmonary artery vegetation on transthoracic echocardiography still remains occasional, as it was evident in our case as well.^7^,^8^

As per the 2018 AHA/ACC Guideline for the Management of Adults with Congenital Heart Disease [COR: I, LOE: C-LD], closure of PDA should be considered in adults having left atrial or ventricular enlargement resulting in a left-to-right shunt without significant pulmonary arterial hypertension i.e. pulmonary arterial pressure being half or less than half of the systemic blood pressure and pulmonary vascular resistance being one-third the peripheral vascular resistance.^9^ Based on a thorough PubMed review, only a few cases of PDA-associated infective endocarditis of pulmonary artery complicated with septic emboli were found. As per our knowledge, the only case of pulmonary artery rupture was presented by Gruber PJ, et al. It was in association with cystic medial degeneration resulting in a pulmonary artery aneurysm that got ruptured, in a 26-years-old pregnant woman.^10^ As to our knowledge, there was no documentation of any case of infective endarteritis associated with perforation of pulmonary artery as in our clinical scenario.

## CONCLUSION

PDA is considered a rare cause of infective endocarditis in the modern era. Even small PDAs noted after one or two years of age should be closed. Our case presents an interesting situation with an unrecognized potentially lethal condition. This required difficult decision making in the surgical approach.

### Authors’ Contributions:

**FM:** Data collection, manuscript writing, editing, figures, revision. **IA:** Idea, critical review. **MAM:** Manuscript writing regarding surgical approach, critical review.
